# Journal of Neonatal Surgery Turns Three

**Published:** 2015-01-10

**Authors:** Yogesh K Sarin, Bilal Mirza

**Affiliations:** 1 Department of Pediatric Surgery, Maulana Azad medical College, University of Delhi, New Delhi, India; 2 Department of Pediatric Surgery, The Children’s Hospital and the Institute of Child Health, Lahore, Pakistan

That Journal of Neonatal Surgery (JNS) has completed its three years of existence is a source of the highest gratification to its founding patrons and editors; is a matter of unbiased congratulation to all those concerned in its inception, interested in its progress, or imbued with a sense of the purposes for which it was established. That it has succeeded beyond the most sanguine expectations of its promoters, no one can gainsay; that it has accomplished the objects that it had in view, i.e., the upholding of publication ethics, the advancement of professional knowledge, and the promotion of good fellowship and good feeling between the members of the fraternity across the borders, no one can controvert; that it will succeed and overcome triumphant all opposing force; that its compulsive course, without retiring ebb, will keep due on the issues published over the past three years most abundantly testify.

We shall continue the same general plan of bringing out the journal issue quarterly, but shall deviate somewhat in detail, as we plan to instantly add on the journal website the accepted manuscripts under the section “Ahead of Publication” without assigning pagination in a sense to disperse latest advances in the specialty without even a minute delay. The coming year 2015 looks promising as we see the light at the end of the tunnel as to the much-awaited indexation with PubMed Central. We look forward to get every manuscript archived on PubMed Central that we have published since the inception of JNS.

## Footnotes

**Source of Support:** Nil

**Conflict of Interest:** None

